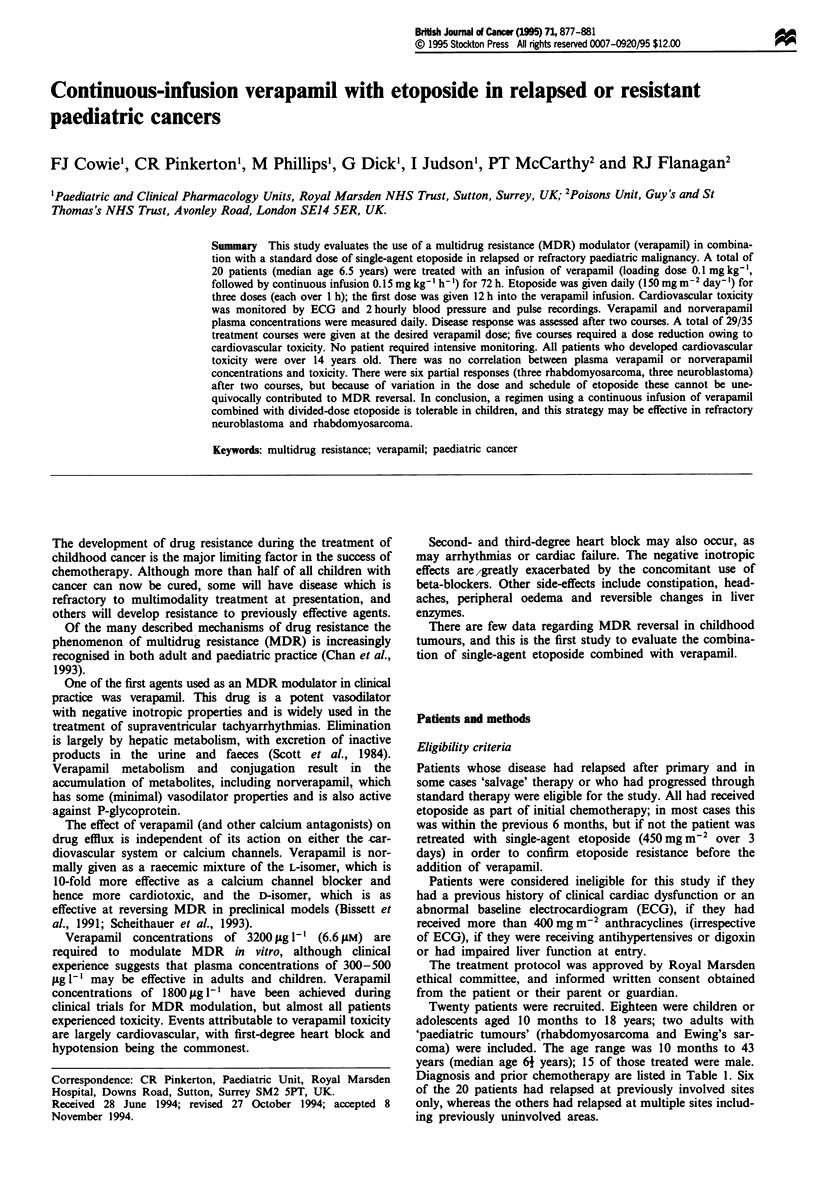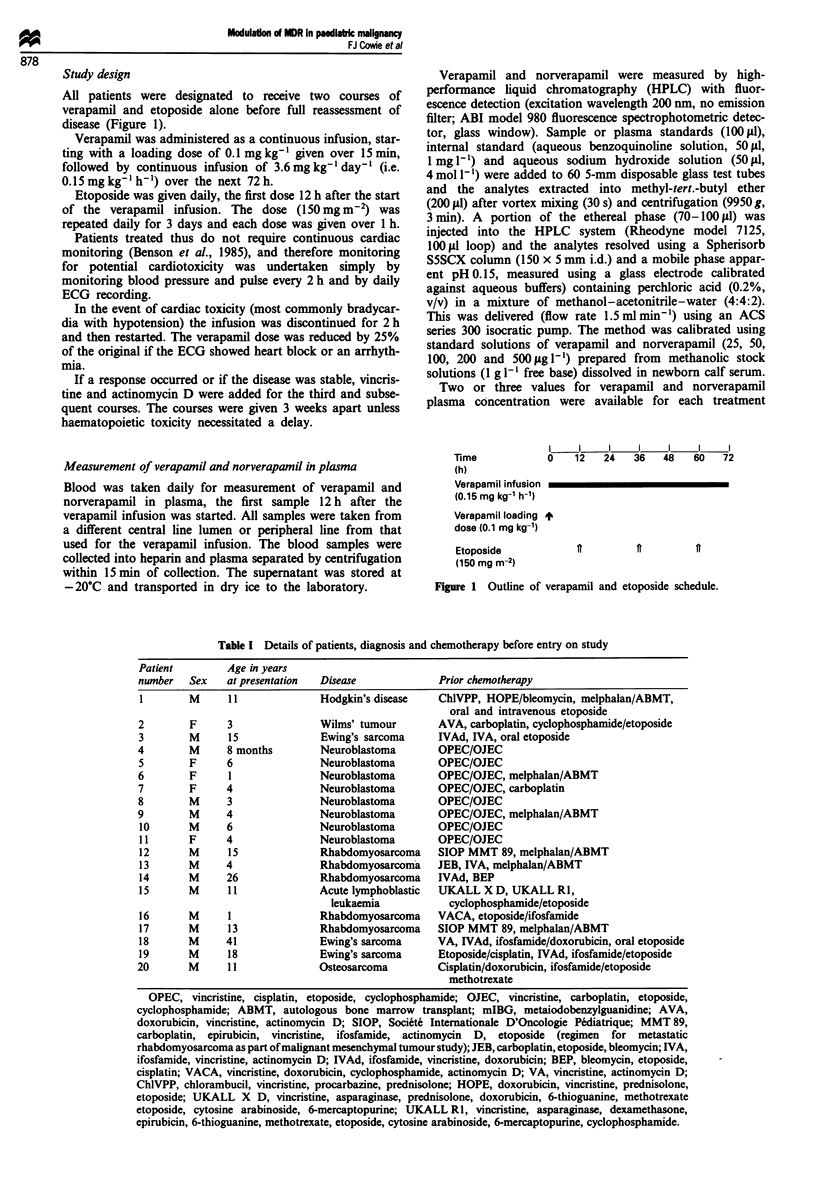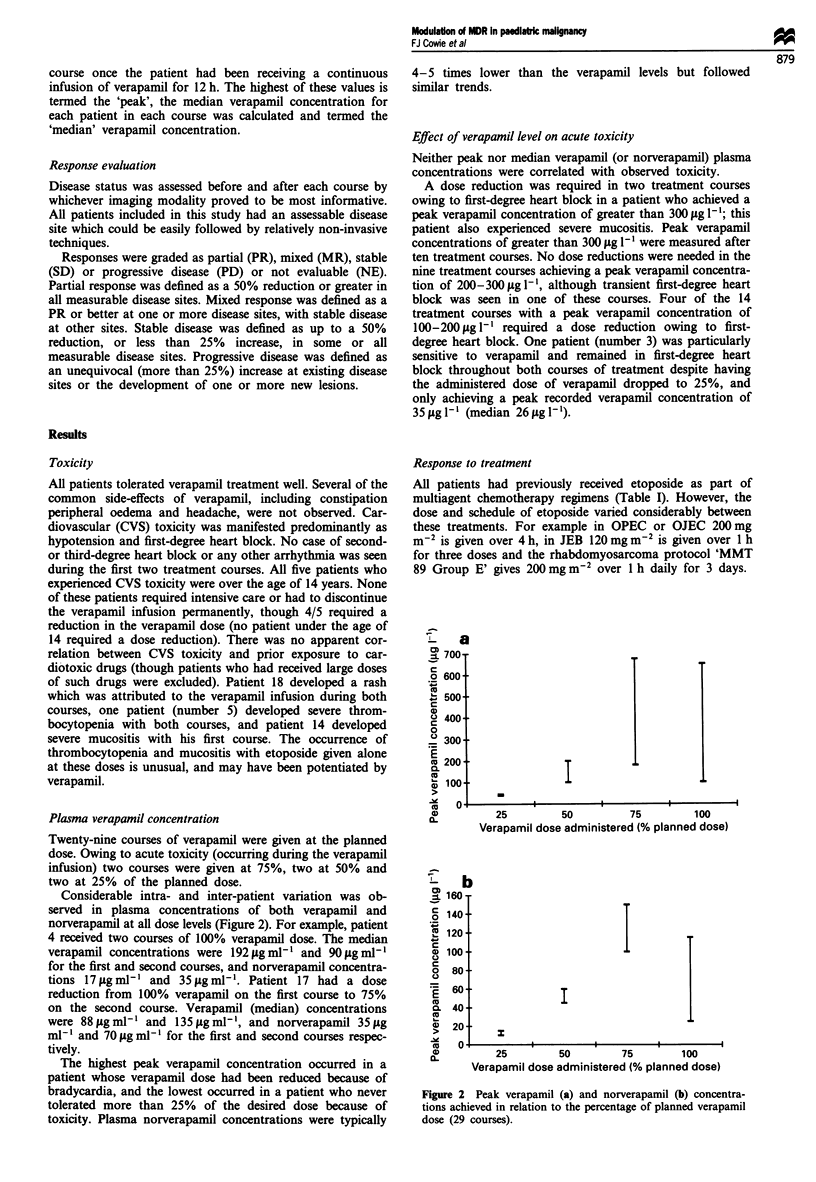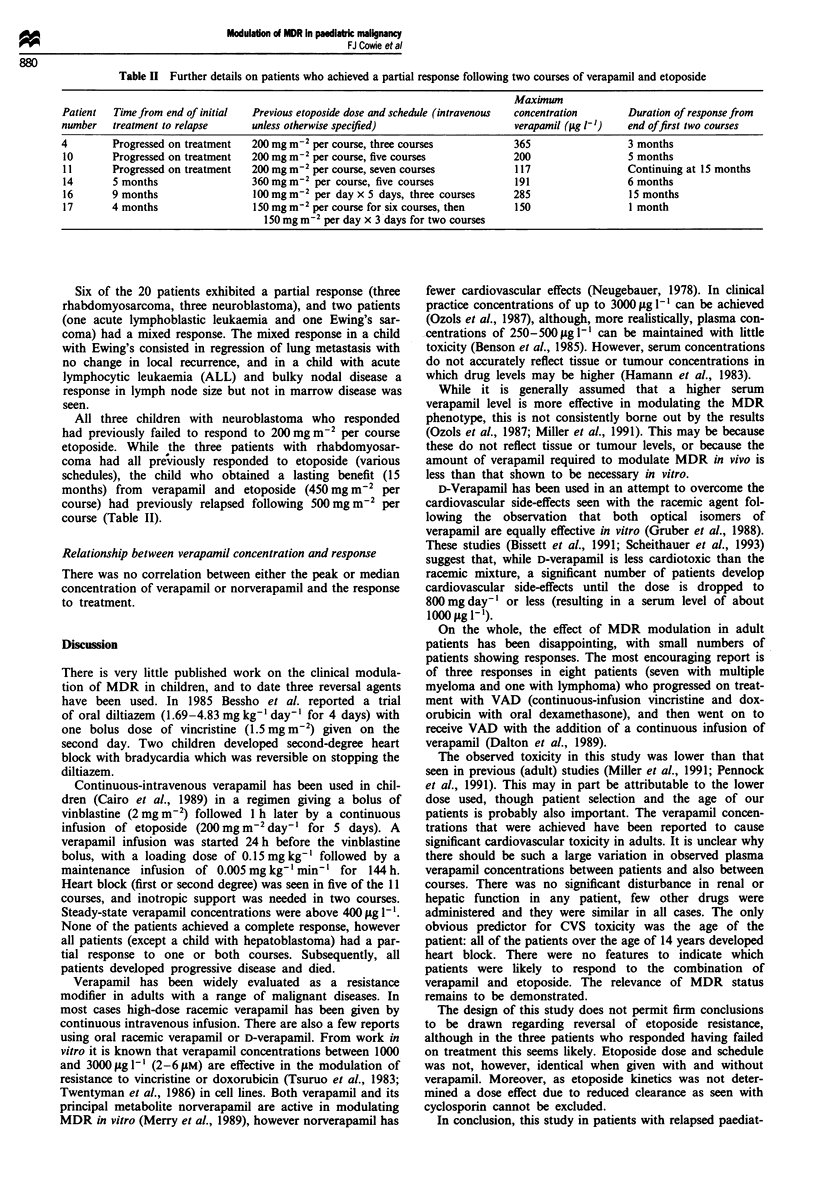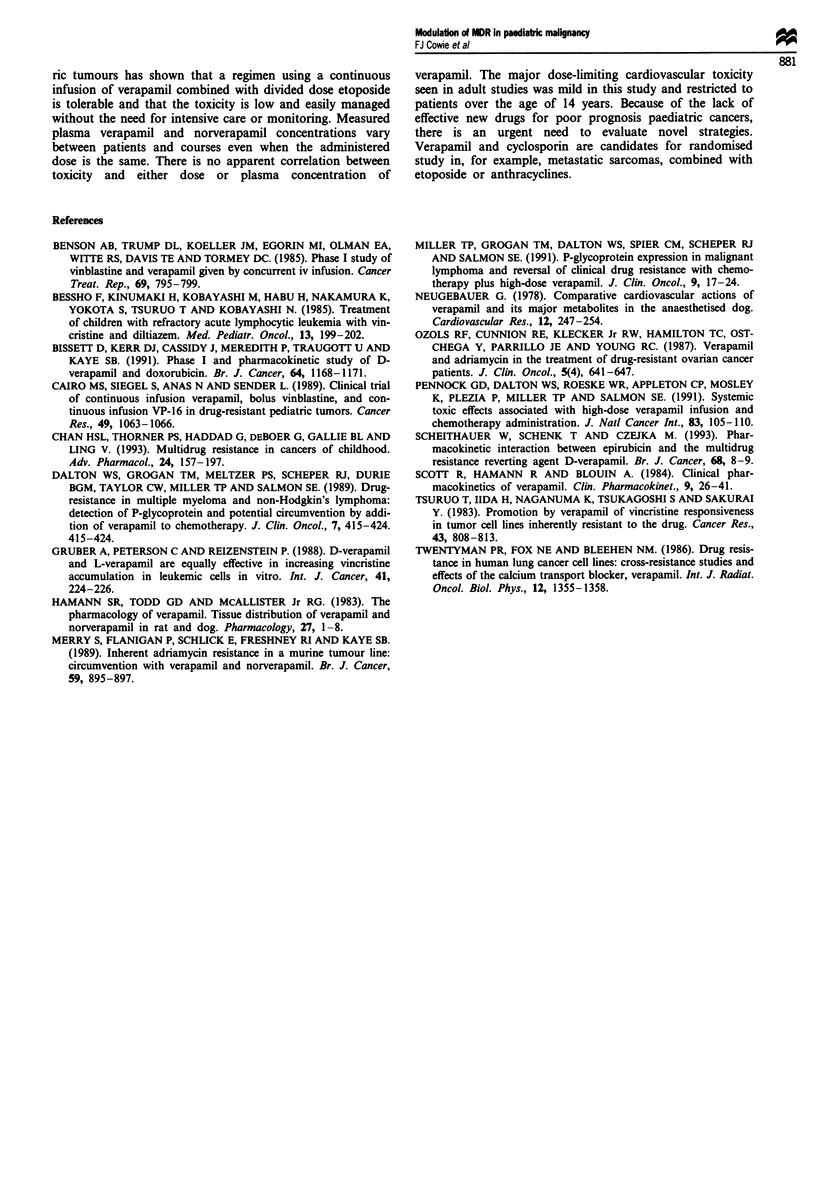# Continuous-infusion verapamil with etoposide in relapsed or resistant paediatric cancers.

**DOI:** 10.1038/bjc.1995.169

**Published:** 1995-04

**Authors:** F. J. Cowie, C. R. Pinkerton, M. Phillips, G. Dick, I. Judson, P. T. McCarthy, R. J. Flanagan

**Affiliations:** Paediatric Unit, Royal Marsden NHS Trust, Sutton, Surrey, UK.

## Abstract

This study evaluates the use of a multidrug resistance (MDR) modulator (verapamil) in combination with a standard dose of single-agent etoposide in relapsed or refractory paediatric malignancy. A total of 20 patients (median age 6.5 years) were treated with an infusion of verapamil (loading dose 0.1 mg kg-1, followed by continuous infusion 0.15 mg kg-1 h-1) for 72 h. Etoposide was given daily (150 mg m-2 day-1) for three doses (each over 1 h); the first dose was given 12 h into the verapamil infusion. Cardiovascular toxicity was monitored by ECG and 2 hourly blood pressure and pulse recordings. Verapamil and norverapamil plasma concentrations were measured daily. Disease response was assessed after two courses. A total of 29/35 treatment courses were given at the desired verapamil dose; five courses required a dose reduction owing to cardiovascular toxicity. No patient required intensive monitoring. All patients who developed cardiovascular toxicity were over 14 years old. There was no correlation between plasma verapamil or norverapamil concentrations and toxicity. There were six partial responses (three rhabdomyosarcoma, three neuroblastoma) after two courses, but because of variation in the dose and schedule of etoposide these cannot be unequivocally contributed to MDR reversal. In conclusion, a regimen using a continuous infusion of verapamil combined with divided-dose etoposide is tolerable in children, and this strategy may be effective in refractory neuroblastoma and rhabdomyosarcoma.


British Journal of Cancer (1995) 71, 877-881
© 1995 Stockton Press All rights reserved 0007-0920/95 $12.00

### Continuous-infusion verapamil with etoposide in relapsed or resistant paediatric cancers

FJ Cowie[1], CR Pinkerton[1], M Phillips[1], G Dick[1], I Judson[1], PT McCarthy[2] and RJ Flanagan[2]

[1]*Paediatric and Clinical Pharmacology Units, Royal Marsden NHS Trust, Sutton, Surrey, UK;* [2]*Poisons Unit, Guy's and St Thomas's NHS Trust, Avonley Road, London SE14 5ER, UK.*

**Summary** This study evaluates the use of a multidrug resistance (MDR) modulator (verapamil) in combination with a standard dose of single-agent etoposide in relapsed or refractory paediatric malignancy. A total of 20 patients (median age 6.5 years) were treated with an infusion of verapamil (loading dose 0.1 mg $kg^{-1}$, followed by continuous infusion 0.15 mg $kg^{-1}$ $h^{-1}$) for 72 h. Etoposide was given daily (150 mg $m^{-2}$ $day^{-1}$) for three doses (each over 1 h); the first dose was given 12 h into the verapamil infusion. Cardiovascular toxicity was monitored by ECG and 2 hourly blood pressure and pulse recordings. Verapamil and norverapamil plasma concentrations were measured daily. Disease response was assessed after two courses. A total of 29/35 treatment courses were given at the desired verapamil dose; five courses required a dose reduction owing to cardiovascular toxicity. No patient required intensive monitoring. All patients who developed cardiovascular toxicity were over 14 years old. There was no correlation between plasma verapamil or norverapamil concentrations and toxicity. There were six partial responses (three rhabdomyosarcoma, three neuroblastoma) after two courses, but because of variation in the dose and schedule of etoposide these cannot be unequivocally contributed to MDR reversal. In conclusion, a regimen using a continuous infusion of verapamil combined with divided-dose etoposide is tolerable in children, and this strategy may be effective in refractory neuroblastoma and rhabdomyosarcoma.

**Keywords**: multidrug resistance; verapamil; paediatric cancer

The development of drug resistance during the treatment of childhood cancer is the major limiting factor in the success of chemotherapy. Although more than half of all children with cancer can now be cured, some will have disease which is refractory to multimodality treatment at presentation, and others will develop resistance to previously effective agents.

Of the many described mechanisms of drug resistance the phenomenon of multidrug resistance (MDR) is increasingly recognised in both adult and paediatric practice (Chan *et al.*, 1993).

One of the first agents used as an MDR modulator in clinical practice was verapamil. This drug is a potent vasodilator with negative inotropic properties and is widely used in the treatment of supraventricular tachyarrhythmias. Elimination is largely by hepatic metabolism, with excretion of inactive products in the urine and faeces (Scott *et al.*, 1984). Verapamil metabolism and conjugation result in the accumulation of metabolites, including norverapamil, which has some (minimal) vasodilator properties and is also active against P-glycoprotein.

The effect of verapamil (and other calcium antagonists) on drug efflux is independent of its action on either the cardiovascular system or calcium channels. Verapamil is normally given as a raecemic mixture of the L-isomer, which is 10-fold more effective as a calcium channel blocker and hence more cardiotoxic, and the D-isomer, which is as effective at reversing MDR in preclinical models (Bissett *et al.*, 1991; Scheithauer *et al.*, 1993).

Verapamil concentrations of 3200 $\mu g\,l^{-1}$ (6.6 $\mu M$) are required to modulate MDR *in vitro*, although clinical experience suggests that plasma concentrations of 300-500 $\mu g\,l^{-1}$ may be effective in adults and children. Verapamil concentrations of 1800 $\mu g\,l^{-1}$ have been achieved during clinical trials for MDR modulation, but almost all patients experienced toxicity. Events attributable to verapamil toxicity are largely cardiovascular, with first-degree heart block and hypotension being the commonest.

Correspondence: CR Pinkerton, Paediatric Unit, Royal Marsden Hospital, Downs Road, Sutton, Surrey SM2 5PT, UK.
Received 28 June 1994; revised 27 October 1994; accepted 8 November 1994.

Second- and third-degree heart block may also occur, as may arrhythmias or cardiac failure. The negative inotropic effects are greatly exacerbated by the concomitant use of beta-blockers. Other side-effects include constipation, headaches, peripheral oedema and reversible changes in liver enzymes.

There are few data regarding MDR reversal in childhood tumours, and this is the first study to evaluate the combination of single-agent etoposide combined with verapamil.

### Patients and methods

### *Eligibility criteria*

Patients whose disease had relapsed after primary and in some cases 'salvage' therapy or who had progressed through standard therapy were eligible for the study. All had received etoposide as part of initial chemotherapy; in most cases this was within the previous 6 months, but if not the patient was retreated with single-agent etoposide (450 mg $m^{-2}$ over 3 days) in order to confirm etoposide resistance before the addition of verapamil.

Patients were considered ineligible for this study if they had a previous history of clinical cardiac dysfunction or an abnormal baseline electrocardiogram (ECG), if they had received more than 400 mg $m^{-2}$ anthracyclines (irrespective of ECG), if they were receiving antihypertensives or digoxin or had impaired liver function at entry.

The treatment protocol was approved by Royal Marsden ethical committee, and informed written consent obtained from the patient or their parent or guardian.

Twenty patients were recruited. Eighteen were children or adolescents aged 10 months to 18 years; two adults with 'paediatric tumours' (rhabdomyosarcoma and Ewing's sarcoma) were included. The age range was 10 months to 43 years (median age 6½ years); 15 of those treated were male. Diagnosis and prior chemotherapy are listed in Table 1. Six of the 20 patients had relapsed at previously involved sites only, whereas the others had relapsed at multiple sites including previously uninvolved areas.

*Study design*

All patients were designated to receive two courses of verapamil and etoposide alone before full reassessment of disease (Figure 1).

Verapamil was administered as a continuous infusion, starting with a loading dose of 0.1 mg $kg^{-1}$ given over 15 min, followed by continuous infusion of 3.6 mg $kg^{-1}$ $day^{-1}$ (i.e. 0.15 mg $kg^{-1}$ $h^{-1}$) over the next 72 h.

Etoposide was given daily, the first dose 12 h after the start of the verapamil infusion. The dose (150 mg $m^{-2}$) was repeated daily for 3 days and each dose was given over 1 h.

Patients treated thus do not require continuous cardiac monitoring (Benson *et al*., 1985), and therefore monitoring for potential cardiotoxicity was undertaken simply by monitoring blood pressure and pulse every 2 h and by daily ECG recording.

In the event of cardiac toxicity (most commonly bradycardia with hypotension) the infusion was discontinued for 2 h and then restarted. The verapamil dose was reduced by 25% of the original if the ECG showed heart block or an arrhythmia.

If a response occurred or if the disease was stable, vincristine and actinomycin D were added for the third and subsequent courses. The courses were given 3 weeks apart unless haematopoietic toxicity necessitated a delay.

*Measurement of verapamil and norverapamil in plasma*

Blood was taken daily for measurement of verapamil and norverapamil in plasma, the first sample 12 h after the verapamil infusion was started. All samples were taken from a different central line lumen or peripheral line from that used for the verapamil infusion. The blood samples were collected into heparin and plasma separated by centrifugation within 15 min of collection. The supernatant was stored at −20°C and transported in dry ice to the laboratory.

Verapamil and norverapamil were measured by high-performance liquid chromatography (HPLC) with fluorescence detection (excitation wavelength 200 nm, no emission filter; ABI model 980 fluorescence spectrophotometric detector, glass window). Sample or plasma standards (100 µl), internal standard (aqueous benzoquinoline solution, 50 µl, 1 mg $l^{-1}$) and aqueous sodium hydroxide solution (50 µl, 4 mol $l^{-1}$) were added to 60 5-mm disposable glass test tubes and the analytes extracted into methyl-*tert*.-butyl ether (200 µl) after vortex mixing (30 s) and centrifugation (9950 *g*, 3 min). A portion of the ethereal phase (70–100 µl) was injected into the HPLC system (Rheodyne model 7125, 100 µl loop) and the analytes resolved using a Spherisorb S5SCX column (150 × 5 mm i.d.) and a mobile phase apparent pH 0.15, measured using a glass electrode calibrated against aqueous buffers) containing perchloric acid (0.2%, v/v) in a mixture of methanol–acetonitrile–water (4:4:2). This was delivered (flow rate 1.5 ml $min^{-1}$) using an ACS series 300 isocratic pump. The method was calibrated using standard solutions of verapamil and norverapamil (25, 50, 100, 200 and 500 µg $l^{-1}$) prepared from methanolic stock solutions (1 g $l^{-1}$ free base) dissolved in newborn calf serum.

Two or three values for verapamil and norverapamil plasma concentration were available for each treatment

**Figure 1** Outline of verapamil and etoposide schedule.

**Table I** Details of patients, diagnosis and chemotherapy before entry on study

| *Patient number* | *Sex* | *Age in years at presentation* | *Disease* | *Prior chemotherapy* |
|---|---|---|---|---|
| 1 | M | 11 | Hodgkin's disease | ChlVPP, HOPE/bleomycin, melphalan/ABMT, oral and intravenous etoposide |
| 2 | F | 3 | Wilms' tumour | AVA, carboplatin, cyclophosphamide/etoposide |
| 3 | M | 15 | Ewing's sarcoma | IVAd, IVA, oral etoposide |
| 4 | M | 8 months | Neuroblastoma | OPEC/OJEC |
| 5 | F | 6 | Neuroblastoma | OPEC/OJEC |
| 6 | F | 1 | Neuroblastoma | OPEC/OJEC, melphalan/ABMT |
| 7 | F | 4 | Neuroblastoma | OPEC/OJEC, carboplatin |
| 8 | M | 3 | Neuroblastoma | OPEC/OJEC |
| 9 | M | 4 | Neuroblastoma | OPEC/OJEC, melphalan/ABMT |
| 10 | M | 6 | Neuroblastoma | OPEC/OJEC |
| 11 | F | 4 | Neuroblastoma | OPEC/OJEC |
| 12 | M | 15 | Rhabdomyosarcoma | SIOP MMT 89, melphalan/ABMT |
| 13 | M | 4 | Rhabdomyosarcoma | JEB, IVA, melphalan/ABMT |
| 14 | M | 26 | Rhabdomyosarcoma | IVAd, BEP |
| 15 | M | 11 | Acute lymphoblastic leukaemia | UKALL X D, UKALL R1, cyclophosphamide/etoposide |
| 16 | M | 1 | Rhabdomyosarcoma | VACA, etoposide/ifosfamide |
| 17 | M | 13 | Rhabdomyosarcoma | SIOP MMT 89, melphalan/ABMT |
| 18 | M | 41 | Ewing's sarcoma | VA, IVAd, ifosfamide/doxorubicin, oral etoposide |
| 19 | M | 18 | Ewing's sarcoma | Etoposide/cisplatin, IVAd, ifosfamide/etoposide |
| 20 | M | 11 | Osteosarcoma | Cisplatin/doxorubicin, ifosfamide/etoposide methotrexate |

OPEC, vincristine, cisplatin, etoposide, cyclophosphamide; OJEC, vincristine, carboplatin, etoposide, cyclophosphamide; ABMT, autologous bone marrow transplant; mIBG, metaiodobenzylguanidine; AVA, doxorubicin, vincristine, actinomycin D; SIOP, Société Internationale D'Oncologie Pédiatrique; MMT 89, carboplatin, epirubicin, vincristine, ifosfamide, actinomycin D, etoposide (regimen for metastatic rhabdomyosarcoma as part of malignant mesenchymal tumour study); JEB, carboplatin, etoposide, bleomycin; IVA, ifosfamide, vincristine, actinomycin D; IVAd, ifosfamide, vincristine, doxorubicin; BEP, bleomycin, etoposide, cisplatin; VACA, vincristine, doxorubicin, cyclophosphamide, actinomycin D; VA, vincristine, actinomycin D; ChlVPP, chlorambucil, vincristine, procarbazine, prednisolone; HOPE, doxorubicin, vincristine, prednisolone, etoposide; UKALL X D, vincristine, asparaginase, prednisolone, doxorubicin, 6-thioguanine, methotrexate etoposide, cytosine arabinoside, 6-mercaptopurine; UKALL R1, vincristine, asparaginase, dexamethasone, epirubicin, 6-thioguanine, methotrexate, etoposide, cytosine arabinoside, 6-mercaptopurine, cyclophosphamide.

course once the patient had been receiving a continuous infusion of verapamil for 12 h. The highest of these values is termed the 'peak', the median verapamil concentration for each patient in each course was calculated and termed the 'median' verapamil concentration.

*Response evaluation*

Disease status was assessed before and after each course by whichever imaging modality proved to be most informative. All patients included in this study had an assessable disease site which could be easily followed by relatively non-invasive techniques.

Responses were graded as partial (PR), mixed (MR), stable (SD) or progressive disease (PD) or not evaluable (NE). Partial response was defined as a 50% reduction or greater in all measurable disease sites. Mixed response was defined as a PR or better at one or more disease sites, with stable disease at other sites. Stable disease was defined as up to a 50% reduction, or less than 25% increase, in some or all measurable disease sites. Progressive disease was defined as an unequivocal (more than 25%) increase at existing disease sites or the development of one or more new lesions.

### Results

*Toxicity*

All patients tolerated verapamil treatment well. Several of the common side-effects of verapamil, including constipation peripheral oedema and headache, were not observed. Cardiovascular (CVS) toxicity was manifested predominantly as hypotension and first-degree heart block. No case of second- or third-degree heart block or any other arrhythmia was seen during the first two treatment courses. All five patients who experienced CVS toxicity were over the age of 14 years. None of these patients required intensive care or had to discontinue the verapamil infusion permanently, though 4/5 required a reduction in the verapamil dose (no patient under the age of 14 required a dose reduction). There was no apparent correlation between CVS toxicity and prior exposure to cardiotoxic drugs (though patients who had received large doses of such drugs were excluded). Patient 18 developed a rash which was attributed to the verapamil infusion during both courses, one patient (number 5) developed severe thrombocytopenia with both courses, and patient 14 developed severe mucositis with his first course. The occurrence of thrombocytopenia and mucositis with etoposide given alone at these doses is unusual, and may have been potentiated by verapamil.

*Plasma verapamil concentration*

Twenty-nine courses of verapamil were given at the planned dose. Owing to acute toxicity (occurring during the verapamil infusion) two courses were given at 75%, two at 50% and two at 25% of the planned dose.

Considerable intra- and inter-patient variation was observed in plasma concentrations of both verapamil and norverapamil at all dose levels (Figure 2). For example, patient 4 received two courses of 100% verapamil dose. The median verapamil concentrations were 192 $\mu g\ ml^{-1}$ and 90 $\mu g\ ml^{-1}$ for the first and second courses, and norverapamil concentrations 17 $\mu g\ ml^{-1}$ and 35 $\mu g\ ml^{-1}$. Patient 17 had a dose reduction from 100% verapamil on the first course to 75% on the second course. Verapamil (median) concentrations were 88 $\mu g\ ml^{-1}$ and 135 $\mu g\ ml^{-1}$, and norverapamil 35 $\mu g\ ml^{-1}$ and 70 $\mu g\ ml^{-1}$ for the first and second courses respectively.

The highest peak verapamil concentration occurred in a patient whose verapamil dose had been reduced because of bradycardia, and the lowest occurred in a patient who never tolerated more than 25% of the desired dose because of toxicity. Plasma norverapamil concentrations were typically 4–5 times lower than the verapamil levels but followed similar trends.

*Effect of verapamil level on acute toxicity*

Neither peak nor median verapamil (or norverapamil) plasma concentrations were correlated with observed toxicity.

A dose reduction was required in two treatment courses owing to first-degree heart block in a patient who achieved a peak verapamil concentration of greater than 300 $\mu g\ l^{-1}$; this patient also experienced severe mucositis. Peak verapamil concentrations of greater than 300 $\mu g\ l^{-1}$ were measured after ten treatment courses. No dose reductions were needed in the nine treatment courses achieving a peak verapamil concentration of 200–300 $\mu g\ l^{-1}$, although transient first-degree heart block was seen in one of these courses. Four of the 14 treatment courses with a peak verapamil concentration of 100–200 $\mu g\ l^{-1}$ required a dose reduction owing to first-degree heart block. One patient (number 3) was particularly sensitive to verapamil and remained in first-degree heart block throughout both courses of treatment despite having the administered dose of verapamil dropped to 25%, and only achieving a peak recorded verapamil concentration of 35 $\mu g\ l^{-1}$ (median 26 $\mu g\ l^{-1}$).

*Response to treatment*

All patients had previously received etoposide as part of multiagent chemotherapy regimens (Table I). However, the dose and schedule of etoposide varied considerably between these treatments. For example in OPEC or OJEC 200 mg $m^{-2}$ is given over 4 h, in JEB 120 mg $m^{-2}$ is given over 1 h for three doses and the rhabdomyosarcoma protocol 'MMT 89 Group E' gives 200 mg $m^{-2}$ over 1 h daily for 3 days.

**Figure 2** Peak verapamil (a) and norverapamil (b) concentrations achieved in relation to the percentage of planned verapamil dose (29 courses).

Table II Further details on patients who achieved a partial response following two courses of verapamil and etoposide

| Patient number | Time from end of initial treatment to relapse | Previous etoposide dose and schedule (intravenous unless otherwise specified) | Maximum concentration verapamil ($\mu g\ l^{-1}$) | Duration of response from end of first two courses |
|---|---|---|---|---|
| 4 | Progressed on treatment | 200 mg $m^{-2}$ per course, three courses | 365 | 3 months |
| 10 | Progressed on treatment | 200 mg $m^{-2}$ per course, five courses | 200 | 5 months |
| 11 | Progressed on treatment | 200 mg $m^{-2}$ per course, seven courses | 117 | Continuing at 15 months |
| 14 | 5 months | 360 mg $m^{-2}$ per course, five courses | 191 | 6 months |
| 16 | 9 months | 100 mg $m^{-2}$ per day × 5 days, three courses | 285 | 15 months |
| 17 | 4 months | 150 mg $m^{-2}$ per course for six courses, then 150 mg $m^{-2}$ per day × 3 days for two courses | 150 | 1 month |

Six of the 20 patients exhibited a partial response (three rhabdomyosarcoma, three neuroblastoma), and two patients (one acute lymphoblastic leukaemia and one Ewing's sarcoma) had a mixed response. The mixed response in a child with Ewing's consisted in regression of lung metastasis with no change in local recurrence, and in a child with acute lymphocytic leukaemia (ALL) and bulky nodal disease a response in lymph node size but not in marrow disease was seen.

All three children with neuroblastoma who responded had previously failed to respond to 200 mg $m^{-2}$ per course etoposide. While the three patients with rhabdomyosarcoma had all previously responded to etoposide (various schedules), the child who obtained a lasting benefit (15 months) from verapamil and etoposide (450 mg $m^{-2}$ per course) had previously relapsed following 500 mg $m^{-2}$ per course (Table II).

*Relationship between verapamil concentration and response*

There was no correlation between either the peak or median concentration of verapamil or norverapamil and the response to treatment.

### Discussion

There is very little published work on the clinical modulation of MDR in children, and to date three reversal agents have been used. In 1985 Bessho *et al.* reported a trial of oral diltiazem (1.69–4.83 mg $kg^{-1}$ $day^{-1}$ for 4 days) with one bolus dose of vincristine (1.5 mg $m^{-2}$) given on the second day. Two children developed second-degree heart block with bradycardia which was reversible on stopping the diltiazem.

Continuous-intravenous verapamil has been used in children (Cairo *et al.*, 1989) in a regimen giving a bolus of vinblastine (2 mg $m^{-2}$) followed 1 h later by a continuous infusion of etoposide (200 mg $m^{-2}$ $day^{-1}$ for 5 days). A verapamil infusion was started 24 h before the vinblastine bolus, with a loading dose of 0.15 mg $kg^{-1}$ followed by a maintenance infusion of 0.005 mg $kg^{-1}$ $min^{-1}$ for 144 h. Heart block (first or second degree) was seen in five of the 11 courses, and inotropic support was needed in two courses. Steady-state verapamil concentrations were above 400 $\mu g\ l^{-1}$. None of the patients achieved a complete response, however all patients (except a child with hepatoblastoma) had a partial response to one or both courses. Subsequently, all patients developed progressive disease and died.

Verapamil has been widely evaluated as a resistance modifier in adults with a range of malignant diseases. In most cases high-dose racemic verapamil has been given by continuous intravenous infusion. There are also a few reports using oral racemic verapamil or D-verapamil. From work *in vitro* it is known that verapamil concentrations between 1000 and 3000 $\mu g\ l^{-1}$ (2–6 $\mu M$) are effective in the modulation of resistance to vincristine or doxorubicin (Tsuruo *et al.*, 1983; Twentyman *et al.*, 1986) in cell lines. Both verapamil and its principal metabolite norverapamil are active in modulating MDR *in vitro* (Merry *et al.*, 1989), however norverapamil has fewer cardiovascular effects (Neugebauer, 1978). In clinical practice concentrations of up to 3000 $\mu g\ l^{-1}$ can be achieved (Ozols *et al.*, 1987), although, more realistically, plasma concentrations of 250–500 $\mu g\ l^{-1}$ can be maintained with little toxicity (Benson *et al.*, 1985). However, serum concentrations do not accurately reflect tissue or tumour concentrations in which drug levels may be higher (Hamann *et al.*, 1983).

While it is generally assumed that a higher serum verapamil level is more effective in modulating the MDR phenotype, this is not consistently borne out by the results (Ozols *et al.*, 1987; Miller *et al.*, 1991). This may be because these do not reflect tissue or tumour levels, or because the amount of verapamil required to modulate MDR *in vivo* is less than that shown to be necessary *in vitro*.

D-Verapamil has been used in an attempt to overcome the cardiovascular side-effects seen with the racemic agent following the observation that both optical isomers of verapamil are equally effective *in vitro* (Gruber *et al.*, 1988). These studies (Bissett *et al.*, 1991; Scheithauer *et al.*, 1993) suggest that, while D-verapamil is less cardiotoxic than the racemic mixture, a significant number of patients develop cardiovascular side-effects until the dose is dropped to 800 mg $day^{-1}$ or less (resulting in a serum level of about 1000 $\mu g\ l^{-1}$).

On the whole, the effect of MDR modulation in adult patients has been disappointing, with small numbers of patients showing responses. The most encouraging report is of three responses in eight patients (seven with multiple myeloma and one with lymphoma) who progressed on treatment with VAD (continuous-infusion vincristine and doxorubicin with oral dexamethasone), and then went on to receive VAD with the addition of a continuous infusion of verapamil (Dalton *et al.*, 1989).

The observed toxicity in this study was lower than that seen in previous (adult) studies (Miller *et al.*, 1991; Pennock *et al.*, 1991). This may in part be attributable to the lower dose used, though patient selection and the age of our patients is probably also important. The verapamil concentrations that were achieved have been reported to cause significant cardiovascular toxicity in adults. It is unclear why there should be such a large variation in observed plasma verapamil concentrations between patients and also between courses. There was no significant disturbance in renal or hepatic function in any patient, few other drugs were administered and they were similar in all cases. The only obvious predictor for CVS toxicity was the age of the patient: all of the patients over the age of 14 years developed heart block. There were no features to indicate which patients were likely to respond to the combination of verapamil and etoposide. The relevance of MDR status remains to be demonstrated.

The design of this study does not permit firm conclusions to be drawn regarding reversal of etoposide resistance, although in the three patients who responded having failed on treatment this seems likely. Etoposide dose and schedule was not, however, identical when given with and without verapamil. Moreover, as etoposide kinetics was not determined a dose effect due to reduced clearance as seen with cyclosporin cannot be excluded.

In conclusion, this study in patients with relapsed paediatric tumours has shown that a regimen using a continuous infusion of verapamil combined with divided dose etoposide is tolerable and that the toxicity is low and easily managed without the need for intensive care or monitoring. Measured plasma verapamil and norverapamil concentrations vary between patients and courses even when the administered dose is the same. There is no apparent correlation between toxicity and either dose or plasma concentration of verapamil. The major dose-limiting cardiovascular toxicity seen in adult studies was mild in this study and restricted to patients over the age of 14 years. Because of the lack of effective new drugs for poor prognosis paediatric cancers, there is an urgent need to evaluate novel strategies. Verapamil and cyclosporin are candidates for randomised study in, for example, metastatic sarcomas, combined with etoposide or anthracyclines.

### References

BENSON AB, TRUMP DL, KOELLER JM, EGORIN MI, OLMAN EA, WITTE RS, DAVIS TE AND TORMEY DC. (1985). Phase I study of vinblastine and verapamil given by concurrent iv infusion. *Cancer Treat. Rep.*, **69**, 795–799.

BESSHO F, KINUMAKI H, KOBAYASHI M, HABU H, NAKAMURA K, YOKOTA S, TSURUO T AND KOBAYASHI N. (1985). Treatment of children with refractory acute lymphocytic leukemia with vincristine and diltiazem. *Med. Pediatr. Oncol.*, **13**, 199–202.

BISSETT D, KERR DJ, CASSIDY J, MEREDITH P, TRAUGOTT U AND KAYE SB. (1991). Phase I and pharmacokinetic study of D-verapamil and doxorubicin. *Br. J. Cancer*, **64**, 1168–1171.

CAIRO MS, SIEGEL S, ANAS N AND SENDER L. (1989). Clinical trial of continuous infusion verapamil, bolus vinblastine, and continuous infusion VP-16 in drug-resistant pediatric tumors. *Cancer Res.*, **49**, 1063–1066.

CHAN HSL, THORNER PS, HADDAD G, DEBOER G, GALLIE BL AND LING V. (1993). Multidrug resistance in cancers of childhood. *Adv. Pharmacol.*, **24**, 157–197.

DALTON WS, GROGAN TM, MELTZER PS, SCHEPER RJ, DURIE BGM, TAYLOR CW, MILLER TP AND SALMON SE. (1989). Drug-resistance in multiple myeloma and non-Hodgkin's lymphoma: detection of P-glycoprotein and potential circumvention by addition of verapamil to chemotherapy. *J. Clin. Oncol.*, **7**, 415–424. 415–424.

GRUBER A, PETERSON C AND REIZENSTEIN P. (1988). D-verapamil and L-verapamil are equally effective in increasing vincristine accumulation in leukemic cells in vitro. *Int. J. Cancer*, **41**, 224–226.

HAMANN SR, TODD GD AND MCALLISTER Jr RG. (1983). The pharmacology of verapamil. Tissue distribution of verapamil and norverapamil in rat and dog. *Pharmacology*, **27**, 1–8.

MERRY S, FLANIGAN P, SCHLICK E, FRESHNEY RI AND KAYE SB. (1989). Inherent adriamycin resistance in a murine tumour line: circumvention with verapamil and norverapamil. *Br. J. Cancer*, **59**, 895–897.

MILLER TP, GROGAN TM, DALTON WS, SPIER CM, SCHEPER RJ AND SALMON SE. (1991). P-glycoprotein expression in malignant lymphoma and reversal of clinical drug resistance with chemotherapy plus high-dose verapamil. *J. Clin. Oncol.*, **9**, 17–24.

NEUGEBAUER G. (1978). Comparative cardiovascular actions of verapamil and its major metabolites in the anaesthetised dog. *Cardiovascular Res.*, **12**, 247–254.

OZOLS RF, CUNNION RE, KLECKER Jr RW, HAMILTON TC, OSTCHEGA Y, PARRILLO JE AND YOUNG RC. (1987). Verapamil and adriamycin in the treatment of drug-resistant ovarian cancer patients. *J. Clin. Oncol.*, **5**(4), 641–647.

PENNOCK GD, DALTON WS, ROESKE WR, APPLETON CP, MOSLEY K, PLEZIA P, MILLER TP AND SALMON SE. (1991). Systemic toxic effects associated with high-dose verapamil infusion and chemotherapy administration. *J. Natl Cancer Int.*, **83**, 105–110.

SCHEITHAUER W, SCHENK T AND CZEJKA M. (1993). Pharmacokinetic interaction between epirubicin and the multidrug resistance reverting agent D-verapamil. *Br. J. Cancer*, **68**, 8–9.

SCOTT R, HAMANN R AND BLOUIN A. (1984). Clinical pharmacokinetics of verapamil. *Clin. Pharmacokinet.*, **9**, 26–41.

TSURUO T, IIDA H, NAGANUMA K, TSUKAGOSHI S AND SAKURAI Y. (1983). Promotion by verapamil of vincristine responsiveness in tumor cell lines inherently resistant to the drug. *Cancer Res.*, **43**, 808–813.

TWENTYMAN PR, FOX NE AND BLEEHEN NM. (1986). Drug resistance in human lung cancer cell lines: cross-resistance studies and effects of the calcium transport blocker, verapamil. *Int. J. Radiat. Oncol. Biol. Phys.*, **12**, 1355–1358.